# Improving detection of differentially expressed gene sets by applying cluster enrichment analysis to Gene Ontology

**DOI:** 10.1186/1471-2105-10-240

**Published:** 2009-08-05

**Authors:** Tao Xu, JianLei Gu, Yan Zhou, LinFang Du

**Affiliations:** 1 College of Life Sciences, Sichuan University, Chengdu 610064, PR China; 2 Shanghai-MOST Key Laboratory of Health and Disease Genomics, Chinese National Human Genome Center at Shanghai, Shanghai 201203, PR China; 3 Department of Microbiology, School of Life Sciences, Fudan University, Shanghai 200433, PR China

## Abstract

**Background:**

Gene set analysis based on Gene Ontology (GO) can be a promising method for the analysis of differential expression patterns. However, current studies that focus on individual GO terms have limited analytical power, because the complex structure of GO introduces strong dependencies among the terms, and some genes that are annotated to a GO term cannot be found by statistically significant enrichment.

**Results:**

We proposed a method for enriching clustered GO terms based on semantic similarity, namely cluster enrichment analysis based on GO (CeaGO), to extend the individual term analysis method. Using an Affymetrix HGU95aV2 chip dataset with simulated gene sets, we illustrated that CeaGO was sensitive enough to detect moderate expression changes. When compared to parent-based individual term analysis methods, the results showed that CeaGO may provide more accurate differentiation of gene expression results. When used with two acute leukemia (ALL and ALL/AML) microarray expression datasets, CeaGO correctly identified specifically enriched GO groups that were overlooked by other individual test methods.

**Conclusion:**

By applying CeaGO to both simulated and real microarray data, we showed that this approach could enhance the interpretation of microarray experiments. CeaGO is currently available at .

## Background

Identifying differentially expressed genes (DEGs) from microarray experiments enables researchers to elucidate related biological processes. In addition to studies focused on individual genes such as SAM[1], statistical techniques have been successfully employed to determine whether predefined groups, for example those in Gene Ontology (GO) [2], or in a metabolic pathway, are differentially expressed. There are two main statistical testing approaches: individual gene analysis (IGA) [3,4] and Gene Set Analysis (GSA) [5]. IGA is performed in two steps: first, genes of interest are selected using a cutoff threshold, and the enriched biological categories are gained by statistically testing these genes against the background: typically all genes in the category (e.g., Fisher's exact test). The major limitation of IGA is that the result is significantly affected by an arbitrarily chosen cutoff in the first step. Hence, the GSA approach was developed to address this issue. GSA methods calculate a score based on all the genes within the gene set. Since it is free of the problems of threshold-based methods, GSA should be more sensitive than IGA, thus identifying gene sets with 'subtle but coordinated' expression changes that cannot be detected by IGA. In previous studies, gene sets were formatted and pre-defined into groups such as independent GO terms and reference KEGG [6] pathways. Little attention was paid to subtle but coordinated expression changes across gene sets or within gene sets. In other words, the structure of gene sets was usually ignored.

Some authors have proposed taking into account the structure of gene sets when testing for gene set enrichment. Draghici *et al*. [7] developed an impact analysis of a signaling pathway that incorporates some crucial factors, such as each gene's position in the given pathway and their interactions. A few studies reported that through an intersect operation on gene sets between different categories, the gene sets could more precisely characterize the biological themes, and more closely represent the true differential expression functions of the data. The SEGS [8] and ADGO [9] methods attempted to improve gene set enrichment analysis by intersecting two different GO categories. Enrichment of GO terms with *p*-values calculated by the IGA method based on its neighbourhood using GO graph topology was also implemented in TopGO [10]. A few attempts [10,11] have been proposed to address the redundant enrichment problem caused by overlapping annotations according to the fact that GO terms within a Directed Acyclic Graph (DAG) represent an inheritance relationship. We abstracted the simplest common opinion of these methods, called the *parent-based *approach which was implemented in the CeaGO package, was to join the genes of children to the parent GO term stepwise, and then tested all the GO terms in the graph topology. *Elim *and *weight *approaches presented in the study of Alexa *et al*. [10] and *parent-child *in Grossmann *et al*. [11]are complicated but useful for minimizing false positives and can enrich GO terms more accurately. Otherwise, if an insufficient number of genes are annotated to one GO term, methods may not be sensitive enough to uncover subtle expression changes, similar to the drawbacks of significance analysis of individual genes [12]. Thus, our goal was to identify some novel expression changes by grouping GO terms. Similarity between pairs of GO terms provided an opportunity to enlarge the GO groups to better interpret the gene expression data.

In this article, we present an effective method, cluster enrichment analysis based on GO (CeaGO), to extend enrichment analysis of individual GO terms and discover significantly clustered GO classes from expression data. A sufficiently rigorous standard could not be found to evaluate the algorithm; instead, a simulation study was performed to assess the statistical properties of this method. Finally, we applied this method to the gene expression profiles of ALL [13] and ALL/AML datasets [14]. The results from both simulated and real data showed that CeaGO is sensitive enough to identify significant expression changes overlooked by individual term tests.

## Methods

**Figure 1:**
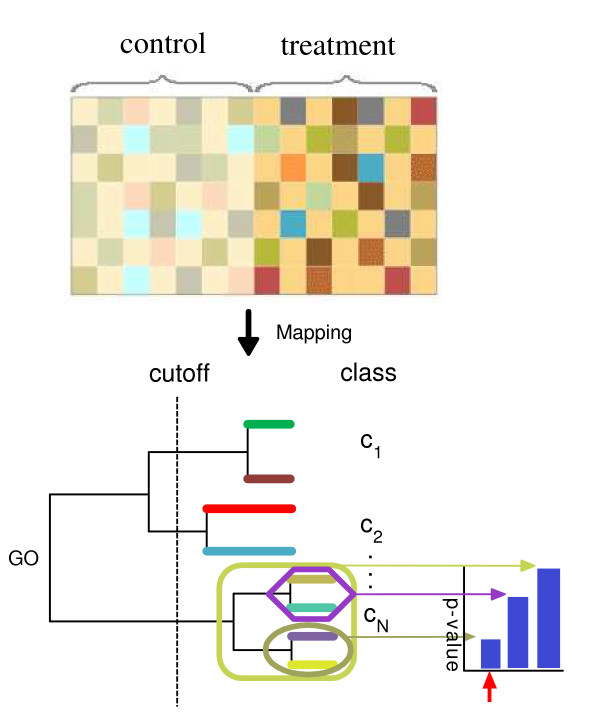
**An overview of the CeaGO method**. The upper panel shows the expression of genes in two classes, for example, gene expression in control versus treatment. After genes are annotated to the GO DAG, a dendrogram is generated using a hierarchical clustering method based on semantic similarity. For such a dendrogram, many classes (*C*_1_, ..., *C*_*N*_) are obtained when the tree meets a user-defined cutoff (lower left panel). The *p*-values are computed according to the gene set enrichment analysis for every subset of each class. The lower right panel shows those *p*-values in a histogram against each subset of the class. The minimum (red arrow) is found and is used to determine the subset (grey green ellipse) of the given class. Such subsets would be assigned as "most likely to be differentially expressed" in the microarray data.

Figure 1 illustrates the overall procedure of CeaGO.

### Semantic similarity calculation

Using groups of genes from microarray experiments, we assigned each gene to the GO terms. For the induced GO graph, we measured the relationship of two GO terms. A variety of algorithms can compute semantic similarity between terms [15-18]. We used the simple but effective measure developed by Resnik [17]. GO allows two terms to share parents. Given all parents (denoted *S(t*_1_, *t*_2_*)*) of two query terms *t*_1 _and *t*_2_, Resnik defined semantic similarity between two GO terms as the information content of the term with minimum probability (denoted *p(t)*) among the common ancestors.

(1)

The GO similarity score can be transformed into a GO distance *d(t*_1_, *t*_2_*)*:

(2)

Note that *S(t) *in this definition is the set of all terms in one ontology.

### Hierarchical clustering

Hierarchical clustering is a multiple step, agglomerative method that sequentially merges samples based on the pair-wise similarity of a given measurement, forming common partitions until all samples are contained in a single group. At each particular stage, the method joins together the two previous clusters that are closest together (most similar). A dendrogram is built by using the semantic similarity metric described above. *N *classes are obtained when the tree meets a user-defined similarity distance threshold (in this study taken as *d *< 2). The model of each class can be described as a vector  = (*G*_1_, *G*_2_, ..., *G*_*n*_), where *G*_*i *_represents the genes annotated to GO terms obtained at the *i*^th ^step in the clustering process. When such a class is formed, various statistical tests can be used to determine whether the genes within the *G*_*i *_showed coordinated expression.

### Gene set enrichment analysis

To detect possibly moderate but coordinated expression changes within a gene set, we employed a simple but robust *Z*-statistic method named PAGE [19] from several available GSA algorithms [12,20-23]. The *Z*-score is calculated according to the following equation:

(3)

Where *μ *is the mean of total fold change [24] values and *σ *is the standard deviation of total fold change values of a given microarray data set, *x *is the mean of the fold change values and *n *is the total number of genes in the gene set. Fold changes are calculated for all genes between two experimental groups (e.g. control versus treatment). *P*-values inferred from *Z*-scores against standard normal distribution are calculated. According to PAGE, 10 samples should be sufficiently close to normal distribution and provide a fairly good statistical test. Therefore, gene sets larger than 10 were used to test the differential gene expression changes.

Next, a vector of significant *p*-values, which was described as  = (*p*_1_, *p*_2_, ..., *p*_*n*_), associated with  was computed for each GO class. If the *p*-values were less than a pre-defined value (e.g. < 0.05), those gene sets were considered as significantly differentially expressed. When such gene sets were found, the gene sets were reduced by retaining the GO subsets with the smallest *p*-values. At this point, the biologically meaningful sets were identified by the tally of GO subsets selected during this step.

The algorithms were implemented in the R programming language . The results were obtained using R version 2.7.1 and the libraries provided by the Bioconductor project , version 2.2.

## Results

### Validation of CeaGO on simulated data

The evaluation of enrichment measurement methods is a challenging task, because biologically meaningful gene sets usually are not known for real datasets. In this study, we introduced an evaluation framework similar to one described previously [10] to address this issue. To imitate real data as closely possible, an artificial data set derived from a HGU95aV2 chip with all 10,503 probes representing genes annotated by terms from the GO biological process subontology was used, and the resulting graph with 4,913 nodes was used as the underlying dataset. Here, we presumed that the expression values of the control and the treatment groups obeyed a standard normal distribution *N*(0, 1). After clustering the GO terms, 188 classes, annotated with more than 10 genes each, were obtained by cutting the dendrogram (*d *< 2). We selected 25 "truly enriched" GO classes at random from the 188 classes. One set was chosen randomly from the subsets in each GO class and denoted as the "truly" differentially expressed gene set. Given those gene sets, *N*(0,1) distributed genes in the treatment groups were replaced by *N*(*μ*,1) distributed genes, with the genes in the control groups remaining as *N*(0,1). The test of dynamic change of *σ *was not performed in this study because *Z*-statistic methods are not sensitive enough to detect changes of sample standard deviation *σ *(see Eq. (3)).

Instead of focusing on a list of differentially expressed groups arbitrarily determined by a pre-defined threshold, we were interested in the groups at the top of the list. Therefore, after enrichment of the GO sets, the enriched groups were sorted in ascending order of their *p*-values. Assuming *S*_*t *_denotes the set of "truly enriched" GO sets, and *E*_*t *_denotes the set of top-scoring GO groups enriched by CeaGO of the same size as *S*_*t*_, an "Exact match" score was used to evaluate the performance of CeaGO:

(4)

The score is the number of pre-selected GO sets found among the top *k *enriched sets. It lies in the interval [0, *k*], where *k *is the perfect prediction by this method.

Another score was also implemented for evaluating the detecting power of the "Possible match" members. Such members represent the fraction of perfect matchings of the pre-selected "truly enriched" GO sets, and the "in but wrong" members that failed, but were still detected as the same class of the "truly enriched" GO sets.

**Figure 2:**
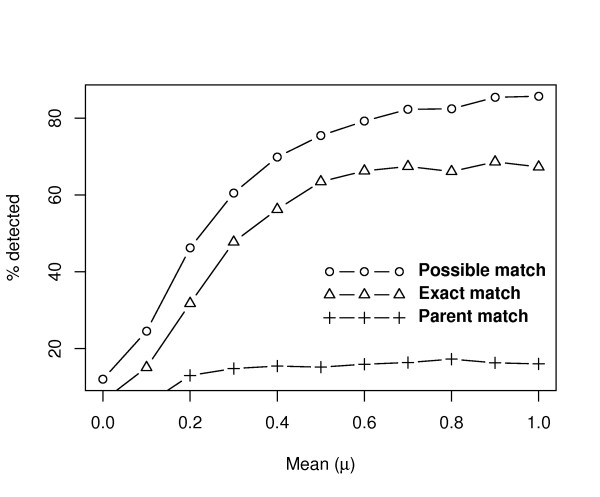
**Effect of changing *μ *on simulated data**. This figure illustrates how results change when the *μ *is changed. "Exact match" represents the percentage of GO groups enriched by the CeaGO exact match to the pre-selected "truly enriched" gene sets. The "Possible match" groups are those that occur in the pre-selected GO classes. The "Parent match" indicates the percentage of top nodes enriched by the *parent-based *enrichment method found among the most recent parent nodes of the pre-selected "truly enriched" GO sets.

The effect of changing *μ *of genes in the treatment groups over the 100 permutations is shown in Figure 2. We observed that detection of both "Possible match" and "Exact match" gene sets exhibited a trend in which larger *μ *had the higher scores. This increasing trend tended to be unclear when the *μ *value was above 0.4. The "Possible match" scores indicated that the PAGE algorithm was sensitive enough to detect differentially expressed groups. The results obtained by this approach showed that most of the "Exact match" gene sets were detectable (about 70%) at a high *μ *(>0.4). About 20% of the "in but wrong" gene sets failed to match the correct GO sets precisely. However, the observed classes might still be helpful in explaining the differentially expressed groups. The "Parent match" scores were calculated to describe the efficacy of CeaGO against the *parent-based *enrichment methods. Only about 15% of GO terms appeared in the pool of most recent parent nodes in 25 pre-selected classes (Figure 2). We have performed the same analysis for one of the *parent-based* methods *Elim *[10]. The results we obtained were similar to those described above. For example, the detecting power of "Parent match" was about 40% above a certain value of *μ *(about 0.6) [see Additional file 1]. These results indicated that the proposed procedure was able to enrich for the correct, differentially expressed gene sets.

### Application of CeaGO to ALL data sets

CeaGO was first applied to the well-known expression dataset Acute Lymphocytic Leukemia (ALL) developed by Chiaretti *et al*. [13]. These data were collected to characterize the relationship between gene expression signatures in ALL-associated cells and genotypic abnormalities in adult patients and the dataset is available from Bioconductor [25]. One use of the dataset has been to examine B-cell lines with this disease and find differential gene expression between the BCR/ABL samples that have rearrangements in the BCR/ABL genes, and NEG samples, which have no evidence of major molecular rearrangements. There are 37 samples for the BCR/ABL group and 42 for the NEG group, each of which has been hybridized to an Affymetrix HGU95Av2 chip containing 12,625 gene-associated probes. We began by normalizing the dataset using the variance stabilizing method VSN [26]. Subsequently, 10,503 genes were successful mapped to GO terms from the BP ontology yielding a list of genes with a GO graph of 3,066 terms.

CeaGO identified five GO clusters with significant *p*-values (<0.05). The raw *p*-values were adjusted using the false discovery rate (FDR) method from Benjamini and Yekutieli [27]. Table 1 presents some novel significant groups that were discarded by individual GO term analysis. For example, genes categorized by *S phase of mitotic cell cycle: 0000084&& regulation of transcription during S-phase of mitotic cell cycle: 0000115 *was significantly differentially expressed in the list scored by CeaGO (*p*-value = 2.9e-5). According to an earlier report [28], the BCR/ABL protein translocates to the nucleus and disrupts an ATR-dependent intra-S phase checkpoint. We observed that genes categorized by *activation of JUN kinase activity: 0007257&& positive regulation of JUN kinase activity: 0043507 *were significantly induced, but neither exhibited significant differential expression using individual GO term analysis. This is consistent with the report that BCR/ABL leukemia oncogene activates Jun kinase and requires Jun for transformation [29]. The accuracy of the enriched GO cluster method for interleukin-6 was also supported by previous research [30].

**Table 1:** Top significant GO groups identified between BCR/ABL and NEG phenotypes for the ALL dataset.

No	GO ID	Term	Rank^a^	*p*-value
1	GO:0043122	regulation of I-kappaB kinase/NF-kappaB ...	3	3.7e-06
	GO:0043123	positive regulation of I-kappaB kinase/N...	-	
	GO:0043124	negative regulation of I-kappaB kinase/N...	-	

2	GO:0000084	S phase of mitotic cell cycle	-	2.9e-05
	GO:0000115	S-phase-specific transcription in mitoti...	-	

3	GO:0032715	negative regulation of interleukin-6 pro...	-	0.012
	GO:0032755	positive regulation of interleukin-6 pro...	-	
	GO:0042226	interleukin-6 biosynthetic process	-	
	GO:0045408	regulation of interleukin-6 biosynthetic...	-	
	GO:0045410	positive regulation of interleukin-6 bio...	-	

4	GO:0032088	inhibition of NF-kappaB transcription fa...	-	0.027
	GO:0043392	negative regulation of DNA binding	-	
	GO:0043433	negative regulation of transcription fac...	-	

5	GO:0007257	activation of JNK activity	-	0.045
	GO:0043507	positive regulation of JNK activity	-	

^a ^Refers to the rank on the list of top GO terms enriched by individual GO term analysis for FDR controlled at 5%. The dash (-) indicates that this GO term was not found on the list generated by individual GO term analysis.

**Figure 3:**
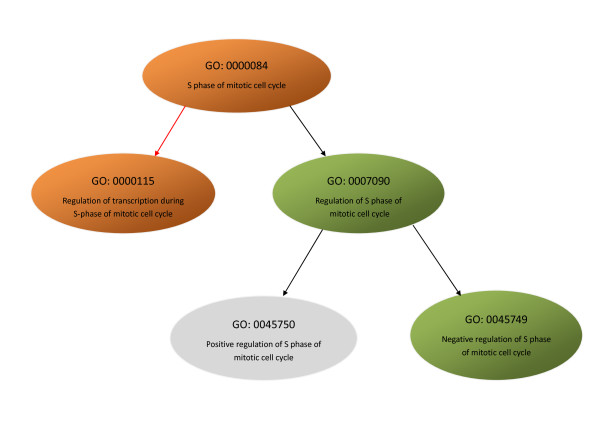
**Subgraph for GO term *S phase of mitotic cell cycle: 0000084***. Orange circles represent significant clusters enriched by CeaGO, green ellipses stand for the annotated GO terms in ALL data but not enriched by CeaGO, and grey ellipse represents ALL genes not mapped to GO terms. Black arrows indicate is-a relationships and red arrow indicates part-of relationship.

The disadvantage with *parent-based *enrichment studies is that they use only the most recent parent node to calculate the significance of gene sets. Therefore, we grouped the GO terms into a cluster, which was a feasible solution for improving the sensitivity of our programme. For example, Figure 3 shows a subgraph induced by *S phase of mitotic cell cycle: 0000084&& regulation of transcription during S-phase of mitotic cell cycle: 0000115 *that exhibited significant differential expression by CeaGO. In this subgraph, the Benjamini and Yekutieli adjusted *p*-value of PAGE analysis of *S phase of mitotic cell cycle: 0000084 *using all genes with GO terms was only 0.025 (*p*-value = 0.0068 in *elim*), which was much less significant than the result of CeaGO analysis (*p*-value = 2.9e-5). Some groups returned similar results to the *parent-based *method, however. For example, *activation of JUN kinase activity: 0007257&& positive regulation of JUN kinase activity: 0043507 *had an adjusted *p*-value of 0.045, while the parent node *positive regulation of JUN kinase activity: 0043507 *had an adjusted *p*-value of 0.038. In these cases, cluster members identified by CeaGO were almost the same as the offspring of the GO term enriched by *parent-based *analysis, which lead to identical significant score. These results indicate that the CeaGO method is more sensitive at detecting certain novel expression changes than *parent-based *enrichment methods, while some coordinated changes were preserved.

### Application of CeaGO to the ALL/AML dataset

The purpose of the present study was to examine the applicability of the CeaGO algorithm. In addition, we tested the algorithm with a published dataset called golubEsets [14], which is also available from Bioconductor. It consists of 7,129 genes from 47 samples of acute lymphoblastic leukemia (ALL), and 25 samples of acute myeloblastic leukemia (AML). Normalization on these samples was also carried out using VSN. This pre-processing resulted in 6,372 genes annotated to GO terms from BP ontology. The induced GO graph contains 2,766 GO terms.

The enriched GO clusters scored by CeaGO are summarized in Table 2. Four clusters had differential gene expression levels in ALL versus AML at the significance level of 0.05. *P*-values were adjusted with the FDR procedure (Benjamini and Yekutieli [27]). CeaGO analysis reported "chemokine" as highly significant from a statistical perspective (*p*-value = 9e-09 for cluster NO. 1, and 3.4e-06 for cluster NO. 2). This is consistent with previous research showing that chemokines affect the proliferation of AML cells and that primary AML cells constitutively release chemokine [31]. Moreover, differences in chemokine responsiveness, as well as chemokine release, are reported to contribute to patient heterogeneity in AML [32]. The second cluster as ranked by the CeaGO analysis concerned "tumor necrosis factor" (FDR corrected *p*-value = 0.0014). The importance of this cluster is well-supported. For example, tumor necrosis factor alpha (TNFα) can increase the proliferation of AML cells [33].

**Table 2:** Top significant GO groups identified between AML and ALL for the ALL/AML dataset.

No	GO ID	Term	Rank^a^	*p*-value
1	GO:0042033	chemokine biosynthetic process	-	9e-09
	GO:0045079	negative regulation of chemokine biosynt...	-	
	GO:0045080	positive regulation of chemokine biosynt...	-	
	GO:0050754	positive regulation of fractalkine biosy...	-	

2	GO:0050927	positive regulation of positive chemotax...	-	3.4e-06
	GO:0050930	induction of positive chemotaxis	-	

3	GO:0032720	negative regulation of tumor necrosis fa...	-	0.0014
	GO:0032760	positive regulation of tumor necrosis fa...	-	
	GO:0042535	positive regulation of tumor necrosis fa...	-	
	GO:0042536	negative regulation of tumor necrosis fa...	-	

4	GO:0045807	positive regulation of endocytosis	-	0.0058
	GO:0048260	positive regulation of receptor-mediated...	-	
	GO:0050766	positive regulation of phagocytosis	-	

^a ^Refers to the rank on the list of the top GO terms enriched by individual GO term analysis for FDR controlled at 5%. The dash (-) indicates that the GO term was not found on the list generated by individual GO term analysis.

## Discussion

Traditional strategies for gene expression analysis have focused on identifying individual genes or pre-defined groups such as those in KEGG pathways and Gene Ontology, which exhibit differences between two states of interest. In this article, we extended expression analysis from prior defined gene sets to a gene set analysis framework that makes use of the structure of GO.

GO has a hierarchical structure that forms a DAG. CeaGO uses the clustering method to combine similar GO terms, rather than using the parent terms, and successfully detected several novel, meaningful categories. One objective of these methods is to enlarge the gene sets to enable identification of new, differentially expressed groups. Methods derived from a parent term with all the genes of its children may introduce unnecessary noise, and fail to detect significant changes. The superior performance of CeaGO than *parent-based *method using PAGE gene set enrichment algorithm is supported by the results presented here on simulated and ALL data. Similar scenarios were observed with the ALL/AML dataset. However, the number of GO groups enriched by the CeaGO method is related to the cutoff of semantic distances. If the threshold was set to the maximum distance of two GO terms of the ontology, CeaGO gave just one cluster, which is too biologically general. If the threshold was set to 0, all GO terms were independent, which is the same as the individual GO term analysis. Fortunately, we found that CeaGO is not sensitive to this parameter, and an arbitrary 20% of the maximum semantic distance was chosen to balance biological meaning and cluster numbers.

The application of CeaGO relies on the accuracy of the gene set test methods. Fortunately, the proposed method operates within a well-defined statistical framework, so that other statistical tests for assessing the significance of GO sets can be used with CeaGO, for example GSEA [12] or GlobalANCOVA [21]. The *Z*-statistic algorithm was employed in this article because of its fast computation advantage over the permutation-based methods. For example, PAGE reduces computation time at least 5,000-fold when performing 5,000 dataset permutations to get a background distribution. However, it suffers from limitations on the normal distribution hypothesis and the minimal size of gene sets. As the accuracy of statistical test methods increases, the performance of CeaGO should improve, as applied to experimental datasets.

In many, if not all cases, analysis with individual GO terms may not be sufficient to reveal changes in specific expression patterns. For example, not all the genes annotated to a "Biological Process" term may exhibit as differentially expressed, but only those with a particular localization such as "membrane" might alter their expression. Through intersection of gene sets between different categories, gene sets can be separated more specifically, facilitating a much more detailed analysis of expression patterns [8,9]. In future studies, we will introduce a comprehensive algorithm to investigate genes within a single GO term or a clustered GO class, with the goal of uncovering the truly differential expression functions in expression data.

## Conclusion

Gene set analysis based on GO is a popular and useful approach to extract biological information from expression data. However, this is limited from showing its full analytical power when only pre-defined GO terms are used, because an insufficient number of genes annotated to one GO term may not be sensitive enough to detect subtle expression changes. Therefore, we developed a novel method to extend the traditional individual GO term analysis. This method dynamically enriches clustered GO terms to identify groups that are significantly differentially expressed in microarray data. Compared to individual GO term analysis (including *parent-based *enrichment methods), the results obtained from both simulated and real microarray data sets showed that the proposed approach is very promising. Furthermore, the CeaGO model can easily be extended to other gene set analysis methods.

## Availability and requirements

Project name: CeaGO

Project homepage: 

Operating system(s): Platform Independent

Programming languages: R and C

## Authors' contributions

TX, YZ and LD jointly designed the CeaGO method. TX implemented the software. TX and JG performed the bioinformatics analyses. TX and YZ drafted the manuscript. All authors read and approved the final manuscript.

## Supplementary Material

Additional file 1 **Effect of changing *μ *on simulated data**. This figure illustrates how results change when the *μ *is changed. "Exact match" represents the percentage of GO groups enriched by the CeaGO exact match to the pre-selected "truly enriched" gene sets. The "Possible match" groups are those that occur in the pre-selected GO classes. The "Parent match" indicates the percentage of top nodes enriched by the *elim *enrichment method found among the most recent parent nodes of the pre-selected "truly enriched" GO sets. Click here for file
