# Supplementary figures and images for: Improving detection of differentially expressed gene sets by applying cluster enrichment analysis to Gene Ontology
Source: BMC Bioinformatics. 2009 Aug 5;10:240. doi: 10.1186/1471-2105-10-240 (PMC2731756; doi:10.1186/1471-2105-10-240)

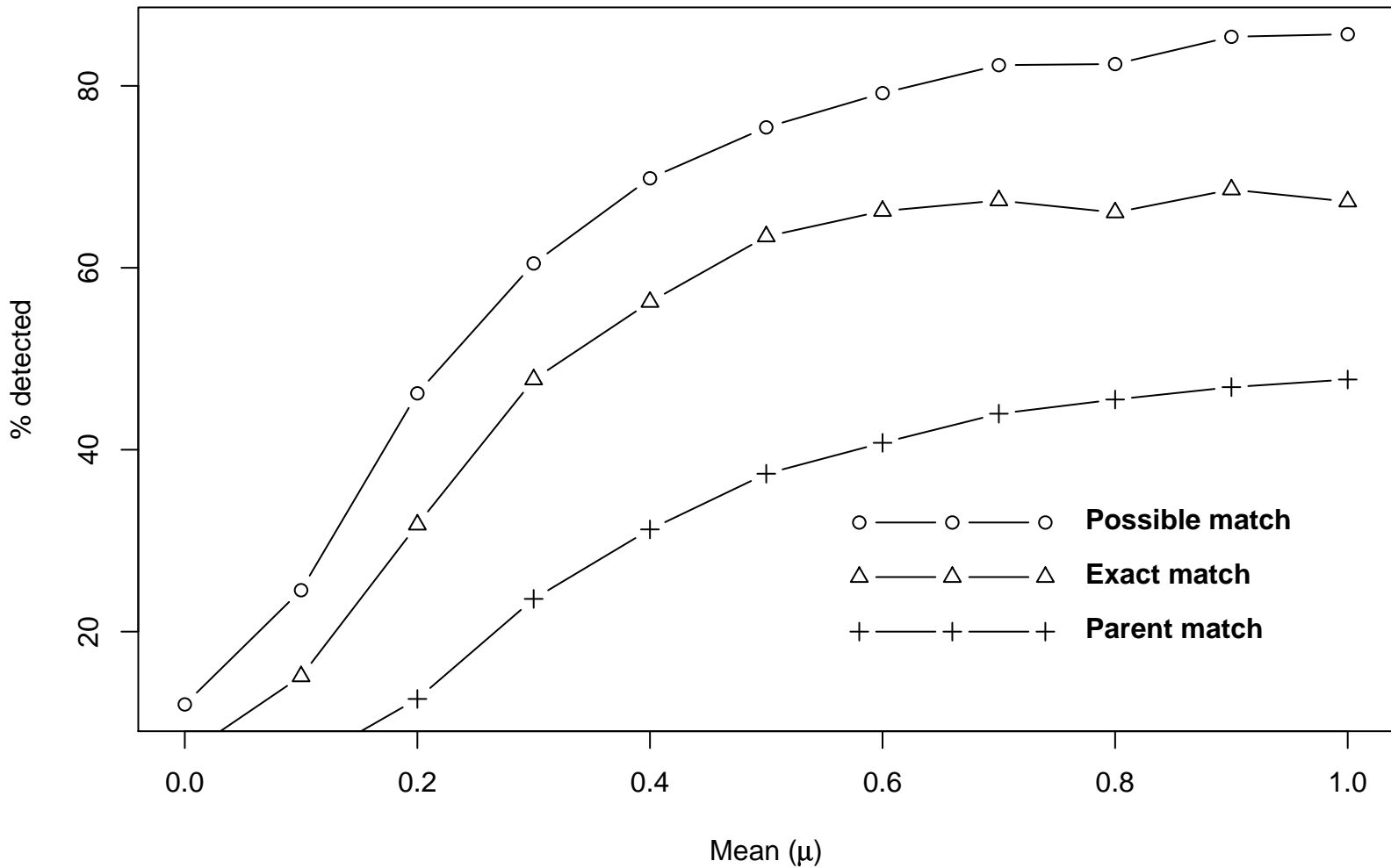

Supplement: Additional file 1 — Effect of changing μ on simulated data. This figure illustrates how results change when the μ is changed. "Exact match" represents the percentage of GO groups enriched by the CeaGO exact match to the pre-selected "truly enriched" gene sets. The "Possible match" groups are those that occur in the pre-selected GO classes. The "Parent match" indicates the percentage of top nodes enriched by the elim enrichment method found among the most recent parent nodes of the pre-selected "truly enriched" GO sets. [file 1471-2105-10-240-S1.pdf]
